# Safety and Efficacy of Polyetheretherketone (PEEK) Cages and Cadaveric Allografts in Transforaminal Lumbar Interbody Fusion (TLIF) for Treating Lumbar Pyogenic Spondylodiscitis

**DOI:** 10.1155/2023/5171620

**Published:** 2023-05-29

**Authors:** Huo-Liang Zheng, Bo Li, Shao-Kuan Song, Peng-Bo Chen, Xin-Feng Zheng, Lei-Sheng Jiang, Sheng-Dan Jiang

**Affiliations:** Department of Clinic of Spine Center, Xinhua Hospital, Shanghai Jiao Tong University School of Medicine, Shanghai 200092, China

## Abstract

**Purpose:**

There have been many studies in the operative management of pyogenic spondylodiscitis with foreign materials. However, it still remains an issue of debate on whether the allografts may be used in pyogenic spondylodiscitis. This study sought to evaluate the safety and effectiveness of PEEK cages and the cadaveric allograft in transforaminal lumbar interbody fusion (TLIF) for treating lumbar pyogenic spondylodiscitis.

**Methods:**

From January 2012 to December 2019, 56 patients underwent surgery for lumbar pyogenic spondylodiscitis. The posterior debridement of all patients and their fusion with allografts, local bone grafts, and bone chip cages were performed before posterior pedicle screw fusion. An assessment of the residual pain, the grade of neurological injury, and the resolution of infection was conducted on 39 patients. The clinical outcome was evaluated using a visual analog scale (VAS) and the Oswestry Disability Index (ODI), and neurological outcomes were appraised based on Frankel grades. The radiological outcomes were evaluated via focal lordosis, lumbar lordosis, and the state of the fusion.

**Results:**

Staphylococcus aureus and Staphylococcus epidermidis were the most common causative organisms. The mean preoperative focal lordosis was −1.2° (−11.4° to 5.7°), and the mean postoperative focal lordosis increased to 10.3° (4.3°–17.2°). At the final follow-up, there were five cases with subsidence of the cage, no case of recurrence, and no case with cage and screw loosening or migration. The mean preoperative VAS and ODI scores were 8.9 and 74.6%, respectively, and improvements in VAS and ODI were 6.6 ± 2.2 and 50.4 ± 21.3%, respectively. The Frankel grade D was found in 10 patients and grade C in 7. Following the final follow-up, only one patient improved from Frankel grade C to grade D while the others recovered completely.

**Conclusion:**

The PEEK cage and cadaveric allograft combined with local bone grafts is a safe and effective choice for intervertebral fusion and restoring sagittal alignment without increased incidence of relapse for treating lumbar pyogenic spondylodiscitis.

## 1. Introduction

Pyogenic spondylitis continues to represent a worldwide problem. Pyogenic spondylitis is relatively rare with an incidence between 0.4 and 2.0 cases per 100,000 each year but can be severe and life-threatening [1, 2]. A conservative strategy, primarily antibiotic therapy and bracing may be effective in managing spondylodiscitis that does not lead to osseous destruction and subsequent instability. Surgical treatment is necessary in more advanced states, especially those with significant instability, deformity, and/or neurological deficits [3]. Today's gold standard of care includes instrumentation of the posterior pedicle screw, radical disc debridement, and intervertebral fusion with titanium cages or autologous bone grafts [4–7].

In terms of the fusion rate, titanium cages are reliable, but the incidence of subsidence and secondary kyphotic deformity remains controversial [5, 6, 8, 9]. Biocompatible polyetheretherketone (PEEK) cages are used extensively in various degenerative spinal diseases as biocompatible alternatives to metal implants. Although some studies have reported that PEEK cages are safe for patients with pyogenic spondylodiscitis [10], the implementation and modification of PEEK cages still need to be further explored.

Local bone grafts from the facet or lamina, however, may be insufficient for satisfied intervertebral fusion in lumbar pyogenic spondylodiscitis. Despite widespread acceptance of tricortical bone grafting for intervertebral fusion, postoperative pain and fracture at the donor site remain serious management issues. Allografts appear to be a promising option, but it still remains an issue of debate whether the allografts could be used in active spinal infection. Few studies have focused on the application of cadaveric allografts and PEEK cages for treating lumbar pyogenic spondylodiscitis. As a consequence, we aimed to determine whether the use of PEEK cages and cadaveric allografts for treating lumbar pyogenic spondylodiscitis was safe and effective.

## 2. Materials and Methods

Fifty-six patients underwent surgery for lumbar pyogenic spondylodiscitis from January 2012 to December 2019, and 39 patients were assessed in this study. The surgical indications included medical treatment failure, severe pain, vertebral destruction resulting in segmental kyphosis, instability, or neurological deficits. All patients were treated with a posterior debridement and fusion with cadaveric allograft and a PEEK cage loaded with bone chips, prior to posterior pedicle screw fixation. In this study, follow-up time for patients averaged 28.3 months, lasting for at least 2 years.

Clinical presentation; imaging findings including X-ray, CT, and MRI; and hematological examinations were used to formulate the diagnosis of lumbar pyogenic spondylodiscitis. An acute spinal hematogenous infection was diagnosed in all patients. Each patient's intraoperative specimens were processed for Gram staining, aerobic and anaerobic culture and sensitivity, fungal culture, and acid-fast staining.

In the surgical strategy, instrumentation of the posterior pedicle screw was followed by intervertebral disc resection, bony debridement, and an intervertebral fusion. A normal saline irrigation was performed after removing the infected tissue from the disc space. Subsequently, cadaveric allografts and local bone grafts were inserted into the disc space, and then, the intervertebral PEEK cage filled with bone chips was implanted obliquely to bridge the endplates in a unilateral TLIF technique. There was no harvesting of the iliac crest bone in any of the patients. Afterwards, a standard single-layer closure was utilized to close the wound.

VAS and ODI were used to evaluate clinical outcomes, while the Frankel scale was used to evaluate neurological results. Postoperatively, lateral X-ray and CT were used to assess the interbody fusion, subsidence, segmental lordosis, and lumbar lordosis. The following criteria were used to determine if fusion was successful: the contiguous bony bridge that connects the instrumented vertebrae, absence of the radiolucency around the cages, and no implant failure. Subsidence is defined as more than 5 mm of sinking of the cage or disc space compared with immediate postoperative imaging. Based on preoperative and follow-up radiographs, segmental lordosis of the fusion level was assessed.

A short period of broad-spectrum intravenous antibiotic therapy was followed by adjustment based on the antibiogram. After surgery, all patients received intravenous antibiotics for a minimum of four weeks, followed by oral antibiotics for at least four weeks or until the CRP and ESR levels returned to normal [11]. In cases without positive cultures, vancomycin and meropenem are given to eliminate both gram-positive and gram-negative bacteria until ESR and CRP levels back to normal.

Patients were evaluated for infection resolution, residual pain, neurological grade, Cobb angle, lumbar lordosis, and fusion and implant statuses at their follow-up visits. Macnab's criteria were used to assess clinical outcome.

SPSS (version 20.0) was used to analyze the data. One-way repeated measure analysis of variance (ANOVA) was used to compare the indicators in the same group at different time points, while the Friedman test was used for data that does not fit Gaussian distribution. We set a statistical significance threshold at less than 0.05.

## 3. Results

In this study, the median patient age was 63 years old, and females predominated (64.1%). All patients had back pain, eight patients (20.5%) had radicular pain, and only seventeen patients (43.6%) had a fever upon presentation. It took an average of 1.8 months (0.5 to 4 months) from symptom onset to diagnosis. There were 14 patients with infection at L4/5 (35.9%), followed by L3/4 in 11 patients (28.2%), L5/S1 in 9 patients (23.1%), and L2/3 in 5 patients (12.8%) (Table 1).

The duration of operation was 106 ± 24.3 minutes. Blood loss during the operation was 205 ± 84.1 ml. Histopathological examination of the intraoperative biopsy confirmed the diagnosis, demonstrating an infiltration of inflammatory cells and vascular proliferation associated with granulation tissue. Bacteria cultures were conducted on all observed specimens taken from the infected site during operation, but only thirty-three positive cultures were found. Staphylococcus aureus was the most common causative organism, detected in twenty-three patients. Of the twenty-three patients, two were positive with methicillin-resistant Staphylococcus aureus. Six patients had Staphylococcus epidermidis, one patient had Streptococcus suis, one patient had Escherichia coli, one patient had Acinetobacter baumannii, and one patient had Corynebacterium (Table 2).

### 3.1. Radiological Outcomes

**Figure 1:**
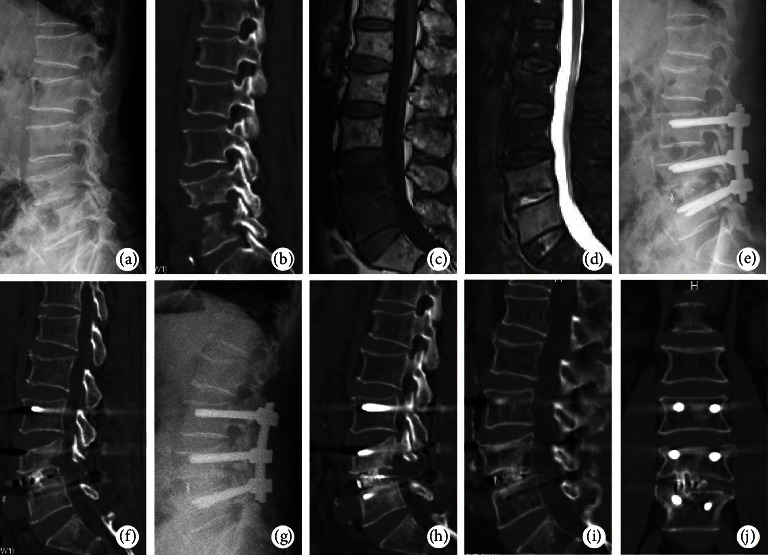
A 68-year-old male with lumbar pyogenic spondylodiscitis. (a) Preoperative lumbar spine X-ray. (b) Preoperative lumbar CT image. (c) T1-weighted image showed hypointensity of L4–L5 vertebral body. (d) T2-weighted fat-suppression sequence showed relative homogeneous enhancement of vertebral bodies and remarkable hyperintensity of the disc. (e, f) Postoperative lumbar radiological images. (g, h) Radiological images at 3 months postoperatively. (i, j) The CT scan showed solid interbody fusion at 1 year after surgery.

The extent of the lesion was determined via preoperative MRI scans. Bony destruction, instability, and deformity were assessed via preoperative X-rays and CT scans. Focal lordosis and lumbar lordosis were measured by using the Cobb method on preoperative and postoperative radiographs. As shown in Table 3, the mean preoperative focal lordosis was −1.2° (−11.4° to 5.7°), and the mean postoperative focal lordosis significantly increased to 10.3° (4.3°–17.2°). The mean two-year postoperative focal lordosis was maintained at 9.8° (4.1°–16.9°). Mean preoperative lumbar lordosis was 21.6° (13.6°–27.5°), and the mean postoperative lumbar lordosis significantly increased to 31.5° (26.1°–37.2°). The mean two-year postoperative lumbar lordosis was maintained at 30.6° (24.9°–36.8°). During the final follow-up, thirty-eight patients had bone bridging across the fusion site, indicating a definitive fusion (Figure 1). At the final follow-up, one patient was suspected to have pseudoarthrosis, five cases showed cage subsidence, and one case showed cage and screw migration or loosening.

### 3.2. Neurological Function

In 17 patients, lower-extremity weakness or sensory changes were noted, although they were rarely severe. Frankel grade D was found in 10 patients and grade C in 7. At the final follow-up, 16 patients recovered completely, while only 1 patient with grade C improved to grade D.

### 3.3. Clinical Outcomes

There was a mean preoperative VAS score of 8.9 and a mean ODI score of 74.6% among all patients. In the two-year follow-up, VAS and ODI scores improved by 6.6 ± 2.2 and 50.4 ± 21.3%, respectively (Table 4).

On the final follow-up, all infections had been cleared. Overall, thirty-three patients (84.6%) had complete pain relief, while six (15.4%) had slight residual pain that did not limit daily activity or need frequent analgesic use. Based on Macnab's criteria [12], thirty-three patients (84.6%) had excellent results, five patients had (12.8%) good results, and only one patient (2.6%) had a fair result.

## 4. Discussion

Although imaging, microbiology, and histopathology techniques have improved, the early diagnosis of pyogenic spondylosis remains challenging. There is always a delay of diagnosis as reported in some studies [13, 14]. Similarly, a median delay of 1.8 months was observed before the correct diagnosis in our series. Two factors might account for this delay of diagnosis. One is that there was no specific symptom in the early stage of lumbar pyogenic spondylodiscitis except for back pain, and the other is that our hospital serves as a tertiary care center for a large region, where patients usually present after failing to receive successful treatment elsewhere. The delay in diagnosis always leads to greater tissue destruction, spinal instability, local kyphotic deformity, and worsening neurological deficits.

Several strategies have been described for the treatment of lumbar pyogenic spondylodiscitis. It was found that autologous bone grafting after debridement proved to be the most efficient and safe method of treating active infections, irrespective of the organism causing the infection, according to Wiltberger in 1952 [15, 16]. Nevertheless, complications, such as pain at the donor site, frequently occur, so grafting with other materials has been introduced. As an additional material, the cadaveric allograft was widely used in treating degenerative spinal disorders, whereas only a minority of the studies reported the cadaveric allograft for interbody fusion in pyogenic spinal infection [17–19]. The application and generalization of the cadaveric allograft in lumbar pyogenic spondylodiscitis still needs to be further confirmed.

Aside from this, some authors claim that grafting with foreign material may reduce antibiotic efficiency and increase the adhesion of bacteria [16, 20]. In comparison to stainless steel, titanium has proven less prone to bacterial colonization [21]. Pee et al. [8] reported the efficacy of titanium cages, titanium mesh cages, and PEEK cages in treating pyogenic spondylodiscitis. They compared clinical and radiological results between patients with pyogenic spondylodiscitis who were treated with cages and struct bone grafts for interbody fusion and found that the struct group had a higher subsidence rate. Cages provided a stronger stability than the struct grafts, which was more favorable for bony fusion. Moreover, studies have shown that PEEK cages do not affect the radiological outcome and increase the risk of reinfection compared to titanium cages [9]. Shiban et al. demonstrated that the use of PEEK cages for interbody fusion is feasible and safe in patients suffering from a pyogenic spinal infection [10]. Similarly, Tschöke et al. [22] proved the efficacy of PEEK interbody cages in treating lumbar pyogenic spondylodiscitis, allowing a stable and solid bony fusion through the posterior TLIF approach. In this study of thirty-nine cases suffering from lumbar pyogenic spondylodiscitis, we reviewed the primary radiological and clinical outcomes. The foreign materials including PEEK and the cadaveric allograft did not seem to affect the clinical outcome and the risk of reinfection. In the present study, we were able to demonstrate that there were no cases of recurring inflammation in patients treated with the PEEK material and allograft after a minimum follow-up of 24 months.

Spinal instrumentation has proven to be safe and effective when used in the presence of active infection [23–25]. Biofilms that bind organisms to implants do not appear to pose any significant clinical risks. According to Hee et al., patients with or without posterior instrumentation had differing outcomes [26]. In comparison to patients who underwent anterior fusion alone, the posterior instrumentation significantly corrected sagittal alignment by 6.2 times (11.1°±7.4° compared with 1.8°±4.6°, *p* = 0.005). Our study also found that the patients treated with additional instrumentation obtained an 11.5° correction of the sagittal alignment postoperatively that was maintained at final follow-up. In comparison with noninstrumented cases, posterior instrumentation provides greater sagittal balance, little loss of correction, and more satisfied fusion rates.

A 97% fusion rate was achieved in our series. Kim et al. [17] reported that 93.3% of patients (14 of 15) using the cadaveric allograft showed osseous union while only 83.3% of patients using the titanium cage showed union. The cadaveric allograft in combination with the PEEK cage may explain the higher fusion rate in our series. On the one hand, the amount of the bone graft for fusion is sufficient because of the cadaveric allograft. On the other hand, the PEEK cage increases the stability and benefits bone fusion.

The PEEK cage can restore and maintain sagittal alignment in treating lumbar pyogenic spondylodiscitis, and the cadaveric allograft can be a useful adjunct for intervertebral fusion. The posterior interbody fusion with the cadaveric allograft and PEEK cage followed by the pedicle screw fixation does not increase relapse rates and is a safe and effective surgical option for treating pyogenic spondylodiscitis.

## Figures and Tables

**Table 1:** Basic data of patients (*x* ± *s*, *n* = 39).

Characteristics	
Age at surgery	63.0 ± 12.7
Sex	
Male	14 (35.9%)
Female	25 (64.1%)
Infected level	
L2/3	5 (12.8%)
L3/4	11 (28.2%)
L4/5	14 (35.9%)
L5/S1	9 (23.1%)
Fever	17 (43.6%)
Operation time (min)	106.0 ± 24.3
Blood loss (ml)	205.0 ± 84.1
Follow-up period (month)	28.3 ± 8.5

**Table 2:** Organisms from specimen culture findings.

Organism	Number of patients
Staphylococcus aureus	21
Methicillin-resistant Staphylococcus aureus	2
Staphylococcus epidermidis	6
Streptococcus suis	1
Escherichia coli	1
Acinetobacter baumannii	1
Corynebacterium	1
Not identified	6

**Table 3:** Focal and lumbar lordosis before and after surgery (*x* ± *s*, *n* = 39).

Parameters	Preoperative	1-month postoperative	2-year postoperative	*p*
Focal lordosis	−1.2 ± 5.3	10.3 ± 3.7^a^	9.8 ± 3.8^a^	<0.001
Lumbar lordosis	21.6 ± 4.3	31.5 ± 3.6^a^	30.6 ± 3.7^a^	<0.001

Note: comparison between preoperative and postoperative parameters; ^a^*p* < 0.05.

**Table 4:** VAS and ODI scores before and after surgery (*x* ± *s*, *n* = 39).

Parameters	Preoperative	1-month postoperative	2-year postoperative	*p*
VAS	8.9 ± 1.1	4.3 ± 1.3^a^	2.3 ± 1.7^a^	<0.001
ODI	74.6 ± 11.8	39.7 ± 9.4^a^	24.2 ± 13.9^a^	<0.001

Note: VAS: visual analog scale; ODI: Oswestry Disability Index. Comparison between preoperative and postoperative parameters; ^a^*p* < 0.05.

## Data Availability

The datasets analyzed in the current study are available from the corresponding authors.
